# Downregulation of Gene Expression by Alpha Satellite Transcripts

**DOI:** 10.3390/ijms262211204

**Published:** 2025-11-20

**Authors:** Sven Ljubić, Maja Matulić, Damir Đermić, Maria Chiara Feliciello, Alfredo Procino, Francesco Passaro, Đurđica Ugarković, Isidoro Feliciello

**Affiliations:** 1 Division of Molecular Biology, Ruder Bošković Institute, Bijenička 54, HR-10000 Zagreb, Croatia; 2 Department of Biology, Faculty of Science, University of Zagreb, HR-10000 Zagreb, Croatia; 3 Department of Statistical Science, Alma Mater Studiorum, University of Bologna, 40126 Bologna, Italy; 4 Department of Clinical Medicine and Surgery, University of Naples Federico II, 80131 Naples, Italy; 5 Division of Urology, Instituto Nazionale Tumori, Fondazione G. Pascale, 80131 Naples, Italy

**Keywords:** satellite DNA, transcription, gene expression, RNA-DNA hybrid, heterochromatin, histone modifications, alpha satellite DNA

## Abstract

Satellite DNAs are highly abundant sequences that build functional centromeres and pericentromeric heterochromatin in many eukaryotes. Apart from this structural role, their involvement in gene expression modulation has been demonstrated, although a detailed understanding of the molecular mechanisms is still lacking. Here, using the major human alpha satellite as a model system, we investigate the role of satellite transcripts in gene expression regulation. We generated cell lines with forced, exogenous overexpression of alpha satellite RNA and followed the expression levels of genes containing alpha satellite repeats within introns. Our results reveal a positive correlation between exogenous alpha satellite expression and the downregulation of alpha-associated genes, strongly suggesting that alpha satellite RNA affects their transcription. Notably, the elevated levels of exogenous alpha satellite RNA did not affect histone modifications characteristic of pericentromeric heterochromatin (e.g., H3K9me3 or H3K18Ac) or euchromatin (e.g., H3K4me2) at intronic alpha satellite loci. We propose that alpha satellite RNA directly interacts with homologous DNA at dispersed intronic satellite loci by forming RNA-DNA hybrid structures, which may affect chromatin structure and transcriptional activity. The results demonstrate that alpha satellite RNA is not only involved in centromere and heterochromatin assembly but, as shown here for the first time, also plays a role in modulating the expression of alpha-associated genes.

## 1. Introduction

Alpha satellite DNA represents a major human satellite that is composed of tandemly arranged, diverged 171 bp long monomers, often organized in complex higher order repeats [1]. The satellite comprises up to 10% of the human genome and is located in the centromeric and pericentromeric regions of all chromosomes in the form of long, Mb-size arrays [2]. In addition to the (peri)centromeric location, a bioinformatic search of the human genome revealed the presence of short arrays of alpha satellite repeats within the euchromatic regions of the genome, including introns and areas near genes [3]. As previously described, one possible origin of these euchromatic, dispersed forms of satellite repeats is due to the molecular mechanism of satellite DNA evolution based on the rolling circle amplification [3,4,5]. This non-canonical occurrence of dispersed satellite repeats has been observed in various species to date and is proposed to be a characteristic feature of all satellite DNAs [6,7,8,9]. In the case of the beetle *Tribolium castaneum*, it was demonstrated that dispersed satellite repeats can influence the expression of neighboring genes upon specific conditions, such as heat stress [10]. In general, heat stress and other kinds of stress, like antibiotic treatment [11], induce transcription of satellite DNA. These transcripts are proposed to guide the deposition of repressive histone marks at homologous satellite sequences, affecting both heterochromatic and euchromatic regions [10,12].

In humans, RNA polymerase II (Pol II) transcribes alpha satellite DNA repeats into long non-coding RNA, and the predominant factors controlling transcription seem to be the presence of centromere–nucleolar contacts [13] and topoisomerase I (TopI) [14]. In addition, a role of N6-methyladenosine (m6A) modification in the regulation of alpha satellite transcription was proposed [15]. Alpha satellite RNA levels fluctuate throughout the cell cycle, peaking in the G2/M phase, and the transcripts are not exported to the cytoplasm [13]. Transcripts of alpha satellite DNA contribute to essential chromosomal functions such as centromere assembly and kinetochore formation [2] and are necessary for proper heterochromatin formation in humans [16,17]. Transcription of alpha satellite DNA is also induced upon heat stress, as shown by studies on different cell lines [18,19]. The increased levels of alpha satellite transcripts after heat stress correlate with the downregulation of genes containing alpha satellite repeats within introns or in the gene vicinity, indicating the possible influence of alpha satellite transcripts on gene expression modulation [19].

To analyze the possible gene-modulatory role of alpha satellite transcripts more deeply and to exclude the effect of heat stress itself or of other stressors on gene expression, we have now developed cell lines with forced, exogenous overexpression of alpha satellite RNA. In such modified cell lines, we monitored the level of expression of genes containing alpha satellite repeats within introns. Although alpha repeats can also be dispersed in the vicinity of genes [3], according to our opinion, the influence of alpha RNA can only be unambiguously established on genes having alpha repeats within the gene body. The results reveal a positive correlation between exogenous alpha satellite expression and downregulation of alpha-associated genes, strongly suggesting an effect of alpha satellite transcripts on gene expression. Furthermore, we performed an analysis of different histone marks on intronic alpha satellite repeats in cells with exogenous expression of alpha satellite RNA, and proposed a possible molecular mechanism by which alpha satellite transcripts modulate gene expression.

## 2. Results

### 2.1. Alpha Satellite Transcription After Transfection

To investigate the possible effect of alpha satellite RNA on gene expression, we constructed vectors expressing alpha satellite monomers in both orientations (171Fw and 171Rev) and monitored their transcription dynamics in the transfected human MJ90-hTERT cell line by RT-qPCR. The exogenous alpha satellite RNA was quantified 24, 48, 72 and 96 h after transfection and compared to its endogenous variant. Primers used for transcriptional analysis of endogenous alpha satellite RNA were able to amplify only tandemly arranged repeats (Figure S1), and since in human pericentromeric heterochromatin, alpha satellite DNA is organized in tandemly arranged monomers [2], it is expected that the primers preferentially recognize transcripts deriving from pericentromeric regions.

**Figure 1:**
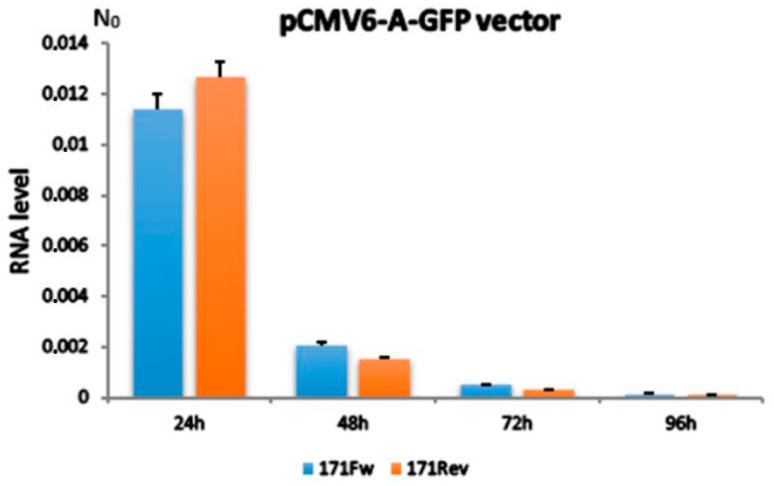
Levels of exogenous alpha satellite RNA expressed from pCMV6-A-GFP vectors 24, 48, 72 and 96 h after transfection. 171Fw indicates the 171 bp alpha satellite monomer insert in forward orientation and 171Rev its inverted form. N_0_ value is expressed in arbitrary fluorescence units and is calculated by taking into account PCR efficiency and baseline fluorescence. Columns show averages of two independent experiments, and error bars indicate standard deviations. No significant difference in expression between vectors with 171Fw and 171Rev, respectively, was observed at any time point (Student’s *t*-test, *p* > 0.1).

The results revealed a significant level of exogenous alpha satellite expression from both 171Fw and 171Rev vectors, the highest being achieved 24 h after transfection and rapidly decreasing afterwards (Figure 1), primarily as a consequence of cell division-related plasmid loss and lack of selective pressure. Additionally, both vector variants demonstrated mutually similar levels of expression during the experiments. Endogenous alpha satellite expression retained its basal levels throughout all investigated time points, indicating standard physiological conditions and lack of stress or toxicity for the cells post-treatment.

### 2.2. Expression Analysis of Alpha-Associated Genes After Transfection

The expression profiles of genes containing dispersed alpha satellite repeats within their intronic regions, described in [19] and listed in Table S1, were explored over the course of four consecutive days, after transfection of the MJ90-hTERT cell line with satellite-expressing vectors, and compared to controls transfected in the same way with unmodified vectors. These included the following: *SLC30A6* (solute carrier family 30 member 6, ID: 55676), *STAM* (signal transducing adapter molecule 1, ID: 8027), *MYO1E* (myosin IE, ID: 4643), *MAP7* (MAP7 domain containing 2, ID: 256714), *ZNF675* (zinc finger protein 675, ID: 171392), *VAV1* (vav guanine nucleotide exchange factor 1, ID: 7409), *PRIM2* (DNA primase subunit 2, ID: 5558) and *DLG2* (discs large homolog 2, ID: 1740). There were no additional genes with targetable intronic alpha satellite segments that met design and expression constraints. This is consistent with the idea that insertion of satellite DNA into euchromatin is generally deleterious and therefore rare, leaving few genes that naturally harbor intronic alpha repeats. The glucuronidase beta gene (*GUSB*, ID: 2990) was used as an endogenous control for expression normalization and a negative control against alpha repeat-associated genes.

**Figure 2:**
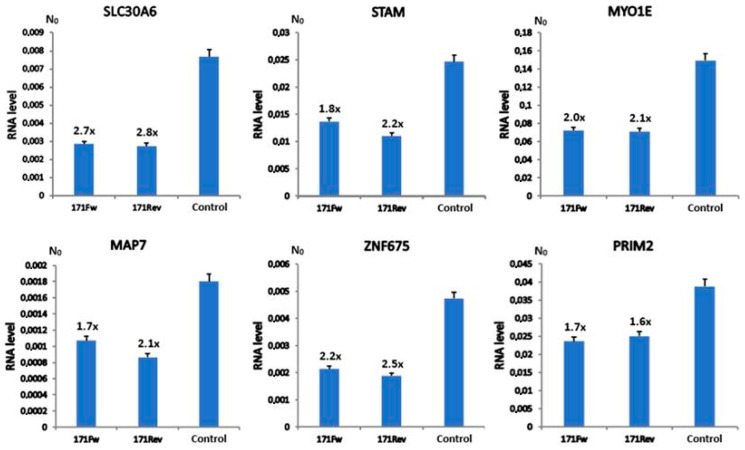
The expression profiles of genes containing alpha satellite repeats within intronic regions, in MJ90hTERT cell lines transfected with satellite-expressing vectors and controls, 24 h after treatment. 171Fw denotes the vector with satellite insert in forward orientation, and 171Rev denotes its inverted counterpart. Control refers to the unaltered pCMV6-A-GFP vector. Gene downregulation rates of transfected samples compared to controls are shown above the error bars, which represent standard deviations. Two independent RT-qPCR experiments were performed, and samples were analyzed in duplicates and averaged mean values are displayed. N_0_ represents the normalized average N_0_ value expressed in arbitrary fluorescence units. *SLC30A6*, *STAM*, *MYO1E*, *MAP7* and *ZNF675* demonstrated the highest level of downregulation between transfected samples and controls (*p* < 10^−3^), and *PRIM2*, although to a lesser extent, was also significant (*p* ≈ 0.02 for each vector, Student’s *t*-test).

Additionally, 24 h post-transfection, a significant downregulation of gene expression was detected for six tested genes relative to controls. These included *SLC30A6*, *STAM*, *MYO1E*, *MAP7*, *ZNF675* and *PRIM2*. Out of those, the first five genes demonstrated the highest level of downregulation between the transfected samples and controls (*p* < 10^−3^); *PRIM2*, although to a lesser extent, was also significant (*p* = 0.02) (Figure 2). Also, there were no observed differences in vectors’ downregulation efficiency between 171Fw and 171Rev transfected cells in all cases, indicating comparable levels of effectiveness regardless of alpha satellite insert orientation. Other investigated time points (48, 72 and 96 h post-transfection) showed no significant differences in gene expression of candidate genes between transfected samples and controls (Figures S2–S4). This is likely due to vector expression dynamics, signifying the most impactful effect of satellite transcription 24 h post-transfection and subsequent rapid decline (Figure 1). The genes *VAV1* and *DLG2* showed no detectable expression in any of the samples at any of the analyzed time points, indicating they are tissue-specific and, therefore, not expressed in the MJ90-hTERT cell line. The *GUSB* gene was stably expressed at all times during the experiments, with no significant variability between samples and controls (Figure S5), confirming its role as a dependable normalization gene for relative RT-qPCR quantification. In addition to *GUSB*, we also tested expression of five more housekeeping genes, *GAPDH*, *TOP3A*, *DEK*, *GPR68* and *IFIT3*, in the MJ90hTERT cell line after 24 h transfection with alpha satellite-expressing vectors (171Fw, 171Rev) and an unmodified control vector. In all cases, no significant differences in gene expression were observed between samples transfected with alpha satellite-expressing vectors and control vector (Figure S6), indicating no influence of alpha satellite RNA on expression of reference genes. It is important to mention that all intronic alpha satellite repeats are flanked by other highly repetitive DNA (*Alu*, L1, SVA, etc.). This prevented the elimination of alpha repeats using CRISPR/Cas9 and enabled the creation of modified cell lines suitable for gene expression studies.

Additionally, it should be noted that our observed results are likely to be understated to a certain degree. The MJ90-hTERT human cell line is quite difficult to transfect effectively without using viral vectors (such as lentivirus), which is often accompanied by higher levels of toxicity and may induce adverse effects for the cells. To avoid these problems, using the lipofection method, we managed to achieve approximately 30–35% transfection efficiency in our experiments by monitoring constitutively expressed GFP inside the cells, an inherent additional trait of our vectors. With higher transfection efficiency, it is likely that the observed downregulatory effects would be more pronounced or even become significant in later time points (48, 72 and 96 h after transfection).

### 2.3. H3K9me3, H3K18ac and H3K4me2 Levels at Alpha Repeats Dispersed Within Genes After Transfection

We analyzed the distribution of silent histone mark H3K9me3, H3K18ac mark characteristic for transcriptional activation of heterochromatin and H3K4me2, typical of open euchromatin, on alpha repeats dispersed within introns of six genes previously tested for expression. Histone marks were analyzed in MJ90hTERT cells 24 h after transfection with 171Fw, 171Rev and unmodified control expression vectors. In order to investigate whether the previously observed downregulation of gene expression could be related to the above-mentioned epigenetic changes, we performed chromatin immunoprecipitation (ChIP) coupled with quantitative real-time PCR, using specific primers for histone modification level analyses of alpha repeats associated with genes (Table S2). A ChIP assay was performed on chromatin isolated from MJ90hTERT cells. The levels of tested histone modifications were measured immediately after a 24 h transfection period and compared to the level of control transfected in the same way with an unmodified vector, using the unpaired *t*-test. In addition, we followed the IgG binding dynamics to investigate loci, and the amount of bound IgG was very low, resulting in a signal below the qPCR threshold.

**Figure 3:**
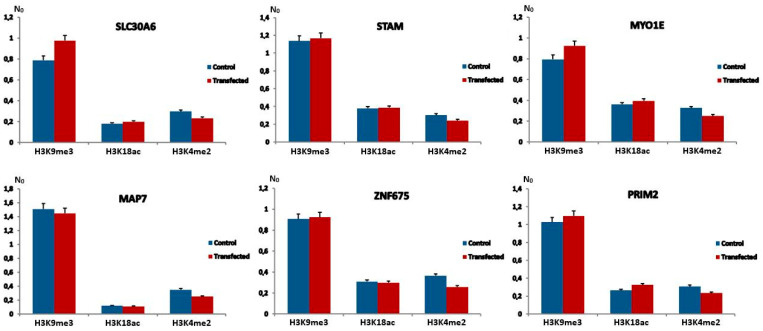
Levels of H3K9me3, H3K18ac and H3K4me2 histone modifications at alpha satellite repeats associated with six genes after 24 h transfection with 171Fw and unmodified control vectors. Levels of histone modifications were measured by ChIP coupled with quantitative real-time PCR on MJ90hTERT chromatin immediately after each treatment. N_0_ values were normalized using N_0_ values of input fractions and represent levels of histone modifications. Columns show averages of two independent experiments, and error bars indicate standard deviations. No significant differences with regard to tested histone modifications at alpha repeats in six genes of interest compared to control samples were observed (*p* > 0.1 in all cases, Student’s *t*-test).

The transfection of MJ90hTERT cells with 171Fw alpha satellite expression vector resulted in no significant differences with regard to tested histone modifications at alpha repeats in six genes of interest compared to control samples, 24 h after treatment (Figure 3). Similarly, no differences were observed after repeated experiments with the 171Rev vector containing an inverted alpha satellite insert (Figure S7). Both vectors showed similar results compared to controls, mirroring their close performance from gene expression analyses (Figure 2). On one hand, it is possible that this outcome is a consequence of the previously mentioned limited transfection potential of MJ90hTERT cells or the lower sensitivity of ChIP experiments. However, it might also be an indicator of other potential mechanism(s) modulating gene expression of alpha satellite repeat-associated genes. Suspecting that the signal originating from individual alpha loci was too weak, we performed ChIP-qPCR on tandemly arranged alpha satellite arrays characteristic of heterochromatin. The results revealed a statistically significant increase in silent histone mark H3K9me3 of 1.5× (*p* < 0.05) in MJ90hTERT cells 24 h after transfection with alpha satellite expression vector (Figure S8). Two other tested histone modifications, H3K18ac and H3K4me2, however, remained unchanged.

### 2.4. Alpha Satellite RNA Level Analysis After RNase H Digestion of RNA:DNA Hybrids

**Figure 4:**
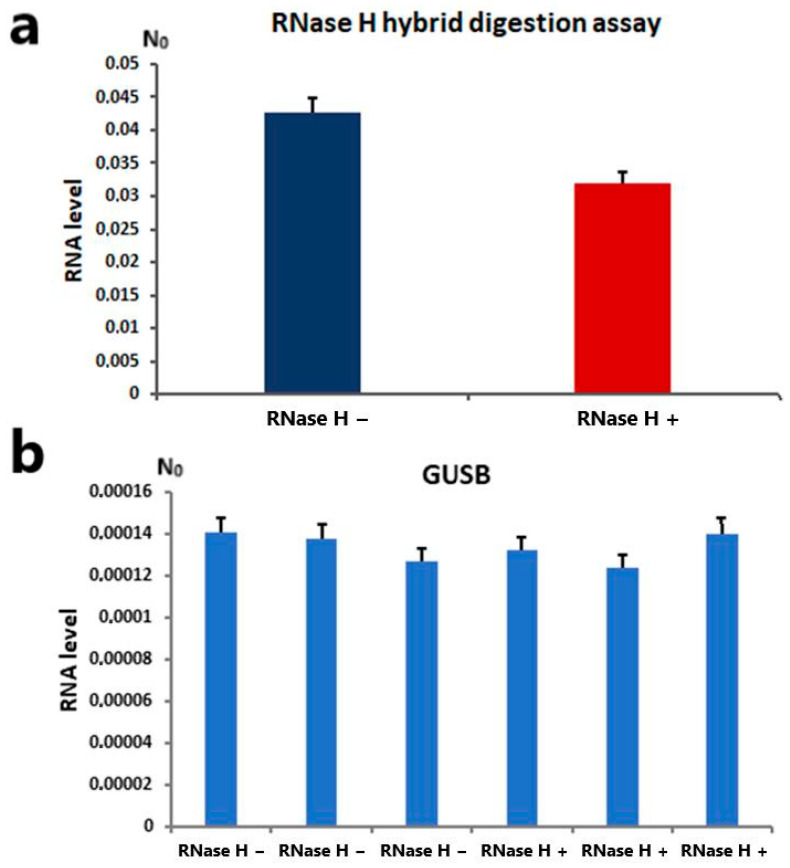
(**a**) Alpha satellite RNA levels in samples treated with RNase H and untreated controls. N_0_ represents normalized average N_0_ values expressed in arbitrary fluorescence units. Columns show averages of two different RT-qPCR experiments performed in triplicate, and error bars represent standard deviations. Statistical significance between controls and treated samples was calculated using Student’s *t*-test (*p* < 0.05). (**b**) Expression profile of housekeeping gene glucuronidase beta (*GUSB*) in MJ90hTERT cell line after treatment with RNase H enzyme and in untreated controls. Three independent RT-qPCR experiments were performed. Error bars represent standard deviations, and averaged N_0_ values are expressed in arbitrary fluorescence units. No significant differences in gene expression were observed between treated samples and controls in all cases (Student’s *t*-test, *p* > 0.1).

To determine whether alpha satellite transcripts actively form RNA:DNA hybrids under standard physiological conditions, RNase H digestion assay was performed. RNase H specifically degrades the RNA strand of an RNA-DNA hybrid. We detected a modest (around 25%) but significant (Student’s *t*-test, *p* < 0.05) reduction in alpha transcripts in samples treated with RNase H compared to untreated controls (Figure 4a). Alpha satellite RNA was quantified by RT-qPCR using the same primer pair as in previous experiments (Fw 5′-CACTCTTTTTGTAGAATCTGC-3′; Rev 5′-AATGCACATATCACTATGTAC-3′). The *GUSB* gene was used as an endogenous housekeeper for normalization and a negative control, being stably and uniformly expressed, showing no significant variation between samples before and after RNAse H treatment (Figure 4b).

## 3. Discussion

While transcription of satellite DNAs is tightly regulated under physiological conditions, upon specific conditions such as heat stress, it is significantly changed and satellite transcripts were proposed to be involved in gene expression modulation [20]. Human satellite III transcription is particularly strongly activated upon heat stress [21], and the transcripts mediate the recruitment of a number of RNA-binding proteins involved in pre-mRNA processing, participating in the control of gene expression at the level of splicing regulation [22,23]. In the case of the major TCAST1 satellite DNA of beetle *Tribolium castaneum*, increased levels of H3K9me2/3 are detected after heat stress at regions of (peri)centromeric heterochromatin and at dispersed satellite repeats and their flanking regions up to 2 kb from the insertion site, indicating that satellite transcripts can act in *trans*, targeting homologous regions in euchromatin. Increased levels of H3K9me2/3 at euchromatic satellite repeats correlate with transient suppression of neighboring genes and indicate the role of TCAST1 satellite siRNAs in the modulation of gene expression [10,12].

In the present study, we transiently transfected MJ90-hTERT fibroblasts with the plasmid expressing alpha satellite DNA monomer and followed the expression of alpha satellite and alpha-associated genes at 24, 48, 72 and 96 h post-transfection. Maximal exogenous expression of alpha satellite exceeding endogenous transcription for more than 10x was obtained 24 h after transfection, coinciding with the downregulation of all alpha-associated genes. The endogenous transcription of alpha satellite was not only significantly lower than the exogenous expression but also remained constant before and after transfection under all tested conditions, indicating that exogenous alpha satellite expression did not affect the endogenous expression regulation. Our present results showed that gene silencing by satellite RNA is not solely mediated by histone modifications but may also involve other forms of transcriptional interference. In humans, ChIP analyses did not reveal significant changes in histone marks typically associated with pericentromeric heterochromatin, such as H3K9me3 and H3K18Ac, nor in euchromatin-associated marks like H3K4me2 at the level of dispersed intronic alpha satellite repeats, although the H3K9me3 level was increased at tandemly arranged alpha repeats. This does not imply a fundamentally different mechanism between *Tribolium* and humans; rather, it suggests an additional silencing pathway alongside the one previously characterized in *Tribolium*. Although ChIP-seq is a powerful technique, conventional protocols often underperform when dealing with low-input material, as in the case of our experiments, where only about 30–35% of cells were efficiently transfected. To conclusively determine whether histone marks also contribute to gene silencing in humans, further experiments using higher-resolution methods are necessary. In particular, single-cell chromatin immunocleavage sequencing (scChIC-seq) may allow the detection of histone modification changes that remain undetectable using conventional bulk ChIP methods [24,25].

**Figure 5:**
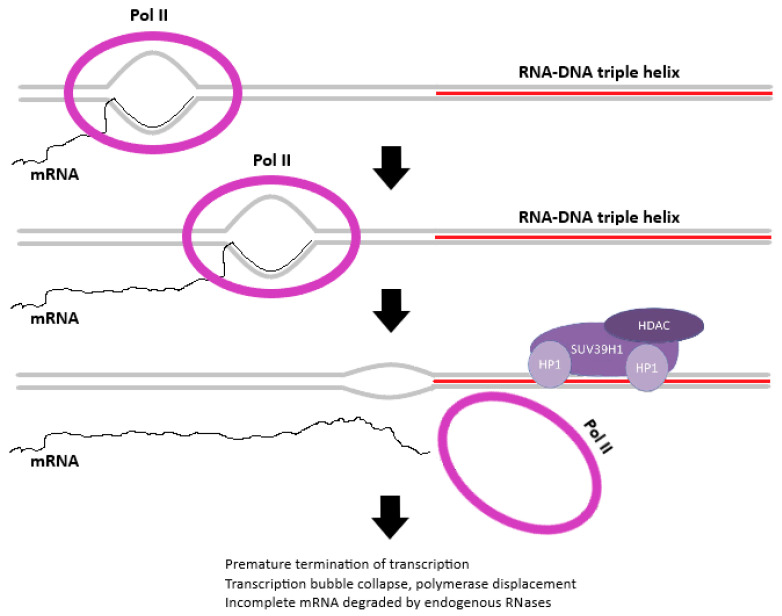
Proposed model of alpha satellite RNA gene-modulatory potential. Depicted is a part of an intronic region of an alpha satellite repeat-associated gene. Termination of transcriptional activity occurs at the boundary between transcriptional machinery and RNA-DNA triplex genomic structure. Pol II signifies eukaryotic RNA Polymerase II enzyme, mRNA is the nascent messenger RNA molecule, and the alpha satellite RNA sequence is highlighted in red. Potentially recruitable RNA-associated chromatin remodeling factors are also displayed (HP1—Heterochromatin Protein 1, SUV39H1—histone methyltransferase, HDAC—histone deacetylase).

Our findings further add a layer of complexity to the mechanisms underlying gene expression downregulation by satellite DNA transcripts. Specifically, the transient expression of exogenous alpha satellite DNAs, produced by transcription from both DNA strands, exerts the same silencing effect on alpha-associated genes, independently of the strand of origin. Based on this observation and on the susceptibility of alpha satellite RNA to RNAse H treatment, we propose that alpha satellite RNA interacts directly with homologous DNA at dispersed intronic satellite loci by forming hybrid structures in the form of triple helices (RNA:DNA:DNA) or R-loops, thereby affecting the transcription of neighboring genes (Figure 5). A mechanism of gene expression modulation based on direct interaction between non-coding RNA and DNA was demonstrated [26]. Unlike classical Watson–Crick base pairing between the strands of the DNA double helix, RNA-DNA hybrids are characterized by Hoogsteen hydrogen bonding between nucleic acid bases. This type of interaction is characterized by a weaker and more flexible association between DNA and RNA molecules, supporting the notion of a mechanism of transient interference based on alpha satellite sequence homology. This could also explain the observed differences in the level of gene suppression among the investigated alpha satellite repeat-associated genes (Figure 2). Given that alpha satellite DNA is polymorphic in nature, with potential monomer sequence variability of up to 45%, and the vector-expressed satellite RNA being a cloned sequence, it is plausible that the observed variability differences in gene repression can be attributed to different degrees of sequence homology. Additionally, the gene-modulatory capabilities of alpha satellite DNA do not depend on gene polarity since similar levels of gene downregulation were observed using either vector, regardless of the direction of transcription (Figure 2). This would imply that the formation of this hybrid genomic structure plays a key role, either directly or by guiding RNA-associated regulatory proteins to specific genomic locations. The formation of triple helices and R-loops seems to be widespread and essential for the regulatory activity of many non-coding RNAs [27,28]. Although many R-loop-forming, long non-coding RNAs act in *cis*, R-loops can also form in *trans*, influencing the expression of protein-coding genes [29]. Triplex formation by long non-coding RNAs was also shown to be able to regulate gene expression in *trans* [28,30]. In mice, pericentromeric satellite DNA transcripts have been shown to form RNA:DNA hybrids that enable the retention of heterochromatin protein 1 (HP1) and the histone methyltransferases SUV39h1 and SUV39h2, which are necessary for heterochromatin formation [17,31]. The observed increase in H3K9me3 level on tandemly arranged alpha satellite arrays characteristic of heterochromatin, after transfection with alpha expression vector, speaks in favor of a potential similar mechanism of recruitment of chromatin modifiers by alpha satellite RNA:DNA hybrids (Figure 5). In order to test whether RNA Pol II accumulates or stalls at around intronic alpha loci, additional experiments using RNA Pol II ChIP should be performed.

In conclusion, our results demonstrate that alpha satellite RNA is not only involved in centromere and heterochromatin assembly, but, for the first time, reveal its role in the modulation of gene expression. Further studies, however, are necessary to reveal the detailed molecular mechanism of gene expression modulation mediated by alpha satellite RNA.

## 4. Materials and Methods

### 4.1. Human Cell Line

Human diploid fibroblast strain MJ90hTERT (HCA2hTERT) was a gift from Dr Olivia M. Pereira-Smith (University of Texas, San Antonio, TX, USA) [32,33,34]. Cells were cultured in appropriate medium (DMEM) supplemented with 10% FBS and 5% CO_2_ at 37 °C.

### 4.2. Construction of Vectors

The alpha satellite 171 bp long monomer was amplified by PCR from MJ90-hTERT genomic DNA and cloned into the pCMV6-A-GFP plasmid vector (OriGene Technologies, Inc., Rockville, MD, USA). A map of the pCMV6-A-GFP plasmid vector is shown in Figure S9. Two separate constructs were created: the first containing the satellite in forward orientation (171Fw) and the second with the insert inverted (171Rev). Inserts were cloned into the vector’s multiple cloning site using modified alpha satellite primers specific for the consensus sequence of 171 bp alpha satellite monomer [35] with inbuilt restriction site sequences. The forward-oriented insert was generated using forward primer (5′-TGCATTGGATCCCATTCTCAGAAACTTCTTTGTG-3′) and reverse primer (5′-TGCATTCTCGAGCTTCTGTCTAGTTTTTATGTGAAG-3′), while the inverted insert was obtained using forward primer (5′-GCATATCTCGAGCATTCTCAGAAACTTCTTTGTG-3′) and reverse primer (5′-TGGCTGGGATCCCTTCTGTCTAGTTTTTATGTGAAG-3′). The unmodified pCMV6-A-GFP vector was used as a negative control. Amplicon fidelity was tested by agarose gel electrophoresis before restriction and ligation into vectors. The JM109 bacterial strain (Promega Corporation, Madison, WI, USA) was subsequently transformed with the above-mentioned recombinant vectors; clones were selected on ampicillin-containing plates, screened by colony PCR, and plasmid vectors were isolated using GenElute HP Plasmid Midiprep Kit (Sigma-Aldrich, Burlington, MA, USA). The sequences of the cloned fragments were validated by Sanger DNA sequencing.

### 4.3. MJ90hTERT Cell Line Transfection

MJ90hTERT (immortalized human skin fibroblasts) cells were transfected with 171Fw, 171Rev and unmodified pCMV6-A-GFP control vector using Lipofectamine 3000 Transfection Reagent (Thermo Fisher Scientific, Waltham, MA, USA), according to the manufacturer’s instructions. Afterwards, cells were incubated for 24, 48, 72 and 96 h at 37 °C in a complete medium.

### 4.4. RNA Isolation and Reverse Transcription

For RNA isolation from cell cultures, the RNeasy Plus Mini Kit (Qiagen, Venlo, Netherlands) was used at each specified time point, and the isolation and reverse transcription were performed using a previously published protocol [11,19]. The specifically modified primer for alpha satellite (Rev 5′-AATGCACATATCACTATGTAC-3′), designed to produce cDNA molecules that differ from genomic DNA in order to avoid DNA contamination, was used [36]. For all samples, negative controls without reverse transcriptase were used.

### 4.5. Quantitative Real-Time PCR (qPCR) Analyses

qPCR analyses were performed 24, 48, 72 and 96 h post-transfection, according to the previously published protocol [10]. Primers used for transcriptional analysis of endogenous alpha satellite RNA were constructed based on consensus sequence derived from cloned alpha satellite monomers of wide-ranging chromosomal origins [35], and the same modified primer used previously in reverse transcription (Rev 5′-AATGCACATATCACTATGTAC-3′) was used in qPCR amplification in order to avoid any potential DNA contamination along with the second primer (Fw 5′-CACTCTTTTTGTAGAATCTGC-3′). Exogenous 171 bp alpha satellite RNA derived from vectors in both orientations was quantified using forward (Fw 5′-CATTCTCAGAAACTTCTTTGTG-3′) or reverse (Rev 5′-CTTCTGTCTAGTTTTTATGTGAAG-3′) primers in combination with vector-specific T7 promoter forward primer (5′-TAATACGACTCACTATAGGG-3′). This strategy ensures the specific amplification of exogenous alpha RNA. Candidate genes of interest with intronic alpha satellite repeats were identified by bioinformatic analyses, as described in [3,19] and the primer combinations used for their expression analysis are listed in Table S3. Glucuronidase beta (*GUSB*) was used as an endogenous control for normalization in human samples as well as a negative control for the aforementioned genes. *GUSB* gene (Gene ID: 2990) was stably expressed at all time points without any variation among samples after transfection. Five additional housekeeping genes were used as negative controls: glyceraldehyde-3-phosphate dehydrogenase (*GAPDH,* ID: 2597), DNA topoisomerase III alpha (*TOP3A*, ID: 7156), DEK proto-oncogene (*DEK*, ID: 7913), G protein-coupled receptor 176 (*GPR68*, ID: 8111) and interferon-induced protein with tetratricopeptide repeats 1 (IFIT3, ID: 3437). Sequences of primers of all reference genes are listed in Table S3. The thermal cycling conditions were described in [11,19]. Post-run data were analyzed using LinRegPCR software v.11.1. [37,38], which enables calculation of the starting concentration of amplicon (“N_0_ value”). The N_0_ value determined for each technical replicate was averaged, and the averaged N_0_ values were divided by the N_0_ values of the endogenous control. Statistical analysis of qPCR data was performed using GraphPad v.6.01, and the normalized N_0_ values were compared using the unpaired *t*-test, which compares the means of two unmatched groups.

### 4.6. Chromatin Immunoprecipitation

Additionally, 24 h after transfection, MJ90hTERT cells were processed according to the published protocol [10,19], with the exception of the sonication step, which was performed 30 times for 30 s on ice, using a high-amplitude sonicator. The antibodies used were as follows: Anti-Histone H3 (tri methyl K9, ab8898, Abcam, Cambridge, UK), Anti-Histone H3 (acetyl K18, Abcam, ab1191), Anti-Histone H3 (di methyl K4, Abcam, ab7766) and IgG (sc2027, Santa Cruz Biotechnology, Inc., Dallas, TX, USA). Binding of the precipitated target was monitored by qPCR using the SYBR Green PCR Master mix (Bio-Rad Laboratories, Hercules, CA, USA). Primers used for H3K9me3, H3K18ac and H3K4me2 level analyses of genes with intronic alpha satellite repeats are listed in Table S2. The N_0_ values were normalized using the N_0_ values of the input fractions.

### 4.7. Alpha Satellite RNA:DNA Hybrid Detection Assay

Approximately 2.5  ×  10^6^ of MJ90hTERT cells per sample were washed in PBS, harvested in medium, transferred into tubes and centrifuged at room temperature for 5 min at 400 g. The pellets were resuspended in 600 μL of Lairds buffer (100 mM Tris pH8.5, 200 mM NaCl, 5 mM EDTA, 0.2% SDS). The samples were sonicated (45 s OFF, 15 s ON, 12 cycles) using a sonicator (high-amplitude), centrifuged for 5 min at 12,000 rpm (4 °C) and the supernatants transferred to new tubes. Total nucleic acids were purified with phenol–chloroform–isoamyl alcohol (A156.3, Roth, Karlsruhe, Germany), precipitated with 2.5 volumes of cold ethanol (96%) and 0.1 volume of 3 M sodium acetate (pH 5.2), and washed with 70% ethanol. The air-dried pellets were resuspended in 50 μL of nuclease-free water. A total of 10 μg of chromatin-associated, phenol/chloroform-isolated nucleic acids per sample was incubated for 30 min at 37 °C with 13 U of RNase H (NEB) in 1× buffer (NEB) in a total volume of 50 μL. The untreated (without RNase H) controls (10 μg) were also incubated in the same way. RNase H-treated and untreated samples were then double-digested with DNase I (Rnase-Free Dnase Set, QIAGEN, Venlo, Netherlands), and total RNA was purified using RNeasy Mini Kit (QIAGEN), according to manufacturer instructions. All nucleic acid quantifications were carried out using a Qubit fluorometer (Invitrogen, Waltham, MA, USA).

## Data Availability

The original contributions presented in the study are included in the article/supplementary material; further inquiries can be directed to the corresponding author(s).

## Supplementary Materials

The following supporting information can be downloaded at https://www.mdpi.com/article/10.3390/ijms262211204/s1.
