# Supplementary material for: Downregulation of Gene Expression by Alpha Satellite Transcripts
Source: Int J Mol Sci. 2025 Nov 20;26(22):11204. doi: 10.3390/ijms262211204 (PMC12653875; doi:10.3390/ijms262211204)
Supplement: Supplementary file 1 [file ijms-26-11204-s001.zip › ijms-3893865-supplementary.pdf]

Table S1. List of alpha satellite repeats dispersed on human chromosomes. Genomic positions of repeats (genome assembly GRCh38/hg38), their composition and similarity to consensus alpha satellite sequence is indicated as well as genes associated with each repeat.

**chr No**	**alpha repeat No**	**Genome position**	**Similarity to alpha satellite consensus/size**	**Associated gene**
**2**	1	32213935-32214051	82%, 0.7 monomer	solute carrier family 30 member 6, (***SLC30A6*** ID: 55676), **intron**
**6**	14	57377750-57378048	70%, 1.7 monomer	DNA primase subunit 2 (***PRIM2*** ID: 5558) **intron**
**10**	18	17653288-17653378	76%, 0.5 monomer	signal transducing adapter molecule 1; (***STAM*** ID: 8027), **intron**
**11**	21	85431074-85431184	85%, 0.7 monomer	discs large homolog 2, (***DLG2*** ID: 1740), **intron**
**15**	25	59219764-59219973	88%, 1.2 monomers	myosin IE (***MYO1E***, ID: 4643), **intron**
**19**	28	6819160-6819369	70%, 1.2 monomers	vav guanine nucleotide exchange factor 1, (***VAV1***, ID: 7409), **intron**
**19**	29	23661142-23661242	86%, 0.6 monomer	zinc finger protein 675, (***ZNF675***, ID: 171392), **intron**
**X**	31	20101282-20101513	71%, 1.3 monomers	MAP7 domain containing 2 (***MAP7***, ID: 256714), **intron**

Table S2. List of primers used for histone modification levels analyses of alpha repeats located within introns of genes, in ChIP-qPCR experiments.

**Gene**	**Primers Fw**	**Primers Rev**
SLC30A6	GCCTCCTGAGTTCAAGCAAC	GCATGGTGCCTCATTCCTAT
STAM	TCCCAGTCCATCGAAACCTA	GAAGCTTCATCACCCTCCAA
MYO1E	CGACATGGGTCCAGTCTGAT	AGGAATCTGGATATGTCTTCCA
MAP7	CGCTAATGCTGAAGACATGC	GAAGTGGAATGGGATCTGAAA
ZNF675	GCTTACCTTGGCTTCTCAAAGT	TAGTAGACACCGGGTTTCACC
VAV1	AGACTCCATCCCCCTCAAAA	TCAAGGTGTCAGTAGGGTTGG
PRIM2	GTTCCTGTAAGGGCTCAACG	AGGCTGCAGTAAGCCATGAT
DLG2	GCACCACACTGGTCTTCC	CTGTATTTCAGCATGAGTGACAG

Table S3. List of primers used for expression analysis of alpha repeat-associated genes and reference genes.

**Gene**	**Primers Fw**	**Primers Rev**
SLC30A6	TGATCTTGCTGGAGCATTTG	AAACATGGGGTGGTGTTGTCT
STAM	AACAAAGGCAGCAGTCAACC	TTGATGGGTTTCACCTTTCC
MYO1E	CTGGGAGGAAAGCAGGGTAA	ACACTTTACTCCTCCCCAGC
MAP7	TTCCTGTTGTGAACTTCGGG	CCTTCCCTTTCCTTGTTGCT
ZNF675	ACACTGCACAGCGGAATTTA	GGGGTTCATTCACCATCTCA
VAV1	TGCTTCAAGTCTCTGGACACCAC	TCTCGGGCGCAGAAGTCATA
PRIM2	TGGACTTAAGTTGGGGTTCG	CAAAGCCTTGGACAGTTTGG
DLG2	TTGCATGTTACTGTGCACTCC	CAGAGGAGAAATATGAGACTGCAA
GUSB	GAAAATACGTGGTTGGAGAGCTCATT	CCGAGTGAAGATCCCCTTTTTA
GAPDH	CCACTCCTCCACCTTTGAC	ACCCTGTTGCTGTAGCCA
TOP3A	TCGACTCTTTAACCACACGG	AGATCTGACCTCTACCACAG
DEK	ATGTGGGTCAGTTCAGTGGC	CCAGAAGGCTTTGGATGCAT
GPR68	CTTCCTCTTCCCCATCTGCC	GCAGGAGGGAGAAGTGGTAG
IFIT3	AGGTTCTCTTGGGCCTGAAA	CCTTGTAGCAGCACTCAATC

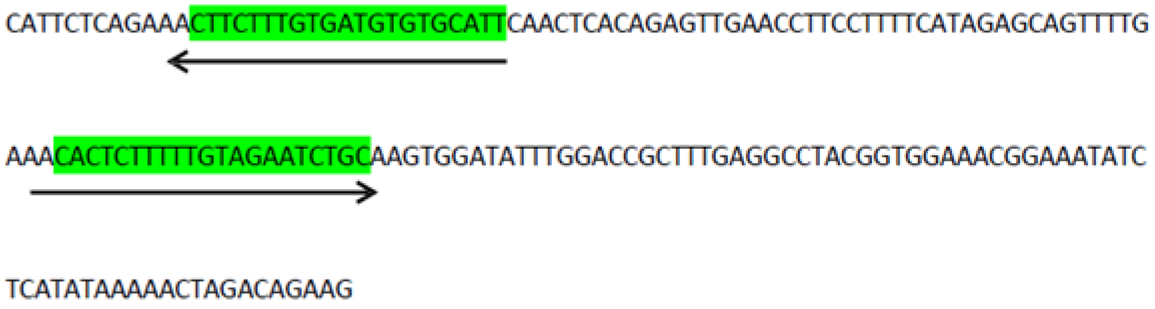

Figure S1. Consensus sequence of 171 bp alpha satellite monomer (Choo et al. 1991) and the annealing positions of primers used in qPCR.

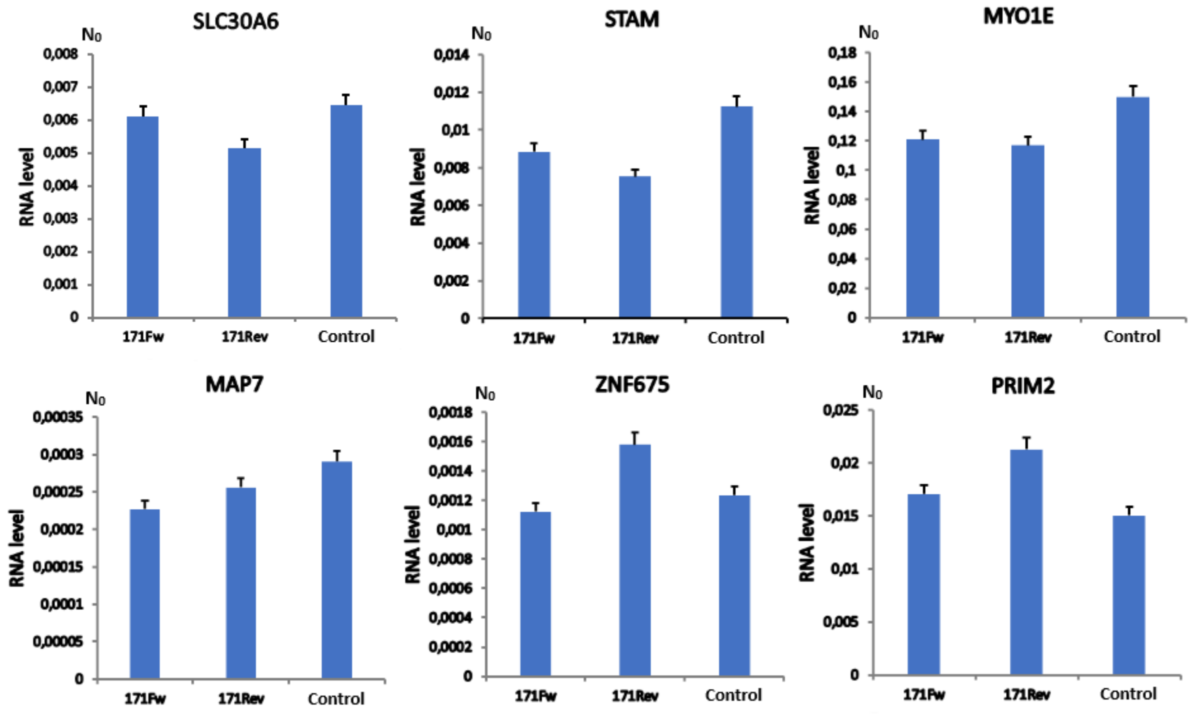

Figure S2. Expression profiles of genes containing alpha satellite repeats within intronic regions, in MJ90hTERT cell lines transfected with satellite expressing vectors and controls, 48 hours after treatment. 171Fw denotes the vector with satellite insert in forward orientation and 171Rev its inverted counterpart. Control refers to unaltered pCMV6-A-GFP vector. Error bars represent standard deviations. N_0 represents normalized average N_0 value expressed in arbitrary fluorescence units. No significant differences in gene expression of candidate genes between transfected samples and controls were observed (Student's t-test, $P>0.05$ in all cases).

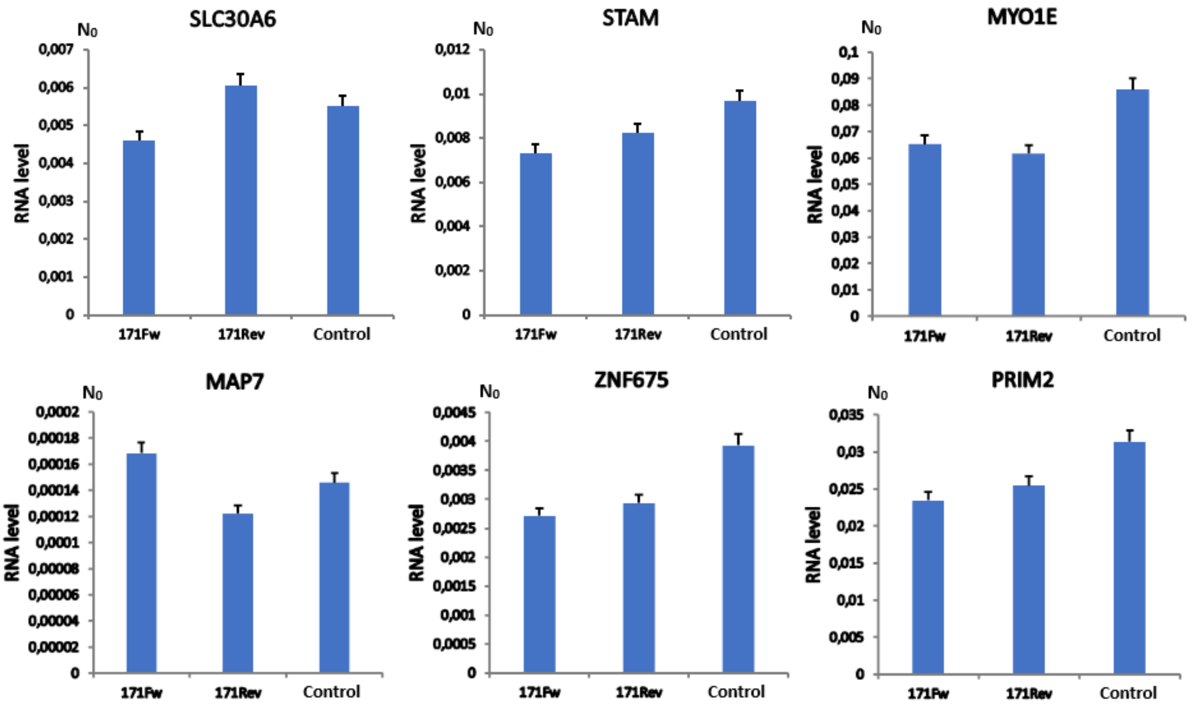

Figure S3. Expression profiles of genes containing alpha satellite repeats within intronic regions, in MJ90hTERT cell lines transfected with satellite expressing vectors and controls, 72 hours after treatment. 171Fw denotes the vector with satellite insert in forward orientation and 171Rev its inverted counterpart. Control refers to unaltered pCMV6-A-GFP vector. Error bars represent standard deviations. N_0 represents normalized average N_0 value expressed in arbitrary fluorescence units. No significant differences in gene expression of candidate genes between transfected samples and controls were observed (Student's t-test, P>0.05 in all cases).

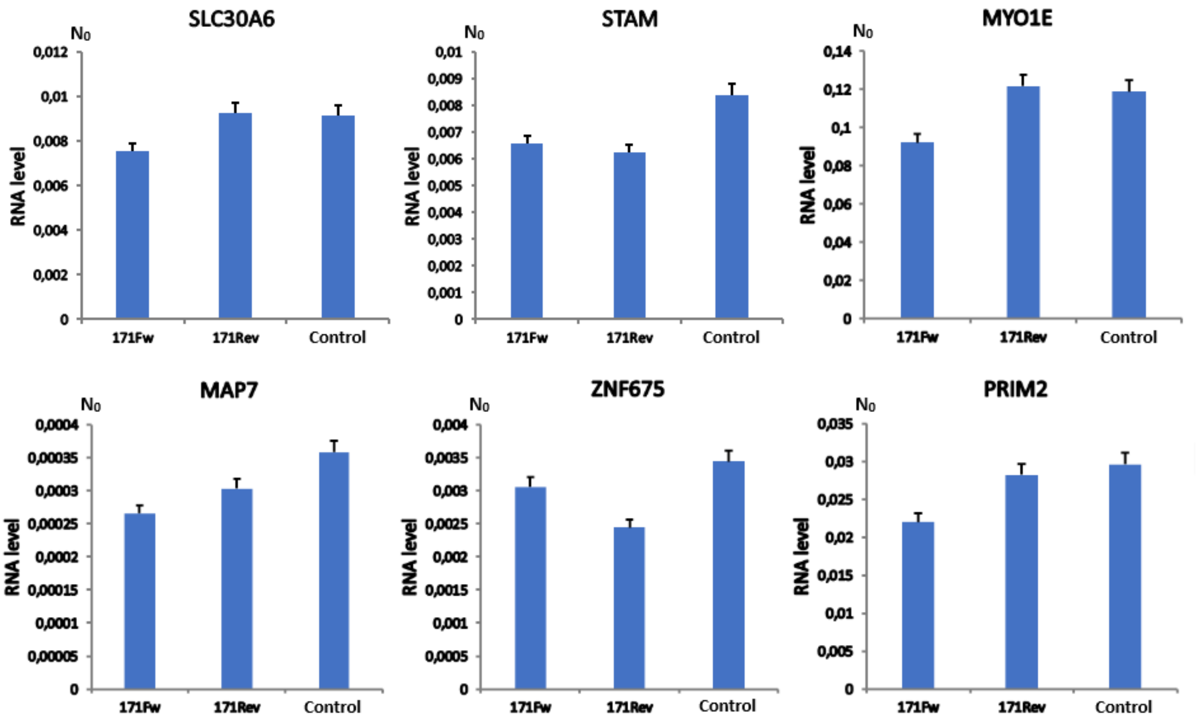

Figure S4. Expression profiles of genes containing alpha satellite repeats within intronic regions, in MJ90hTERT cell lines transfected with satellite expressing vectors and controls, 96 hours after treatment. 171Fw denotes the vector with satellite insert in forward orientation and 171Rev its inverted counterpart. Control refers to unaltered pCMV6-A-GFP vector. Error bars represent standard deviations. N_0 represents normalized average N_0 value expressed in arbitrary fluorescence units. No significant differences in gene expression of candidate genes between transfected samples and controls were observed (Student's t-test, P>0.05 in all cases).

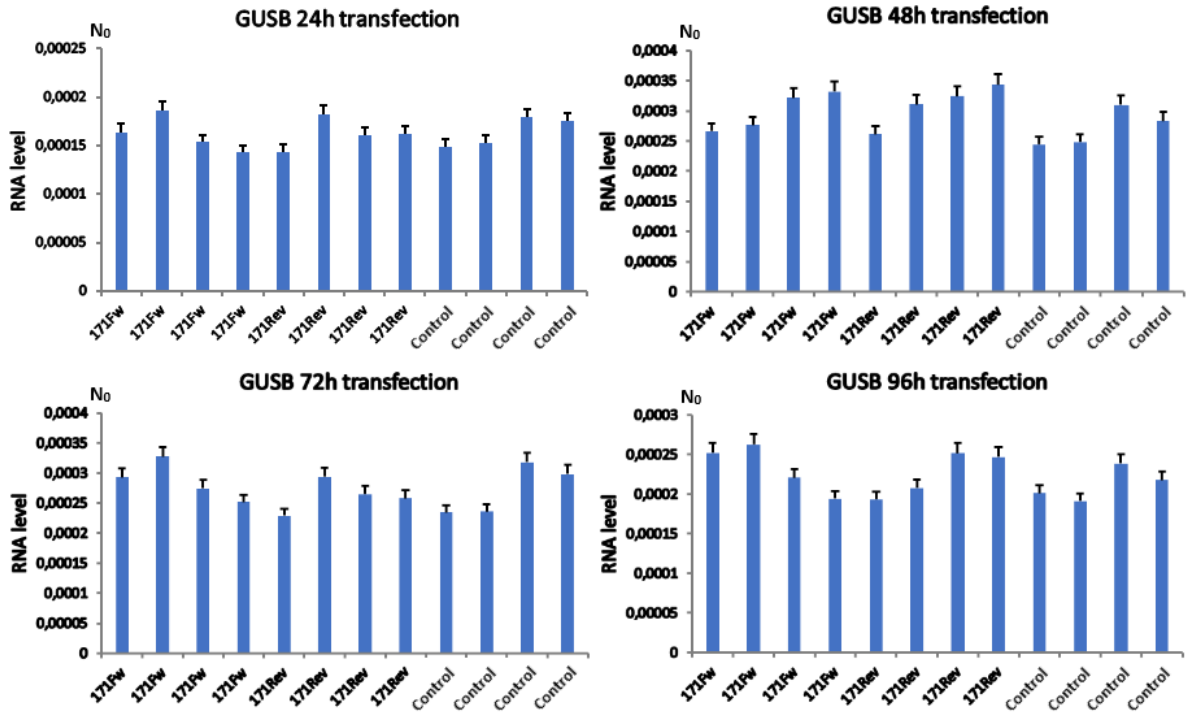

Figure S5. Glucuronidase beta gene expression profile in MJ90hTERT cell line 24, 48, 72 and 96 hours after transfection. Each experiments has been repeated four time. 171Fw denotes the vector with satellite insert in forward orientation and 171Rev its inverted counterpart. Control refers to unaltered pCMV6-A-GFP vector. Error bars represent standard deviations. N_0 values are expressed in arbitrary fluorescence units.

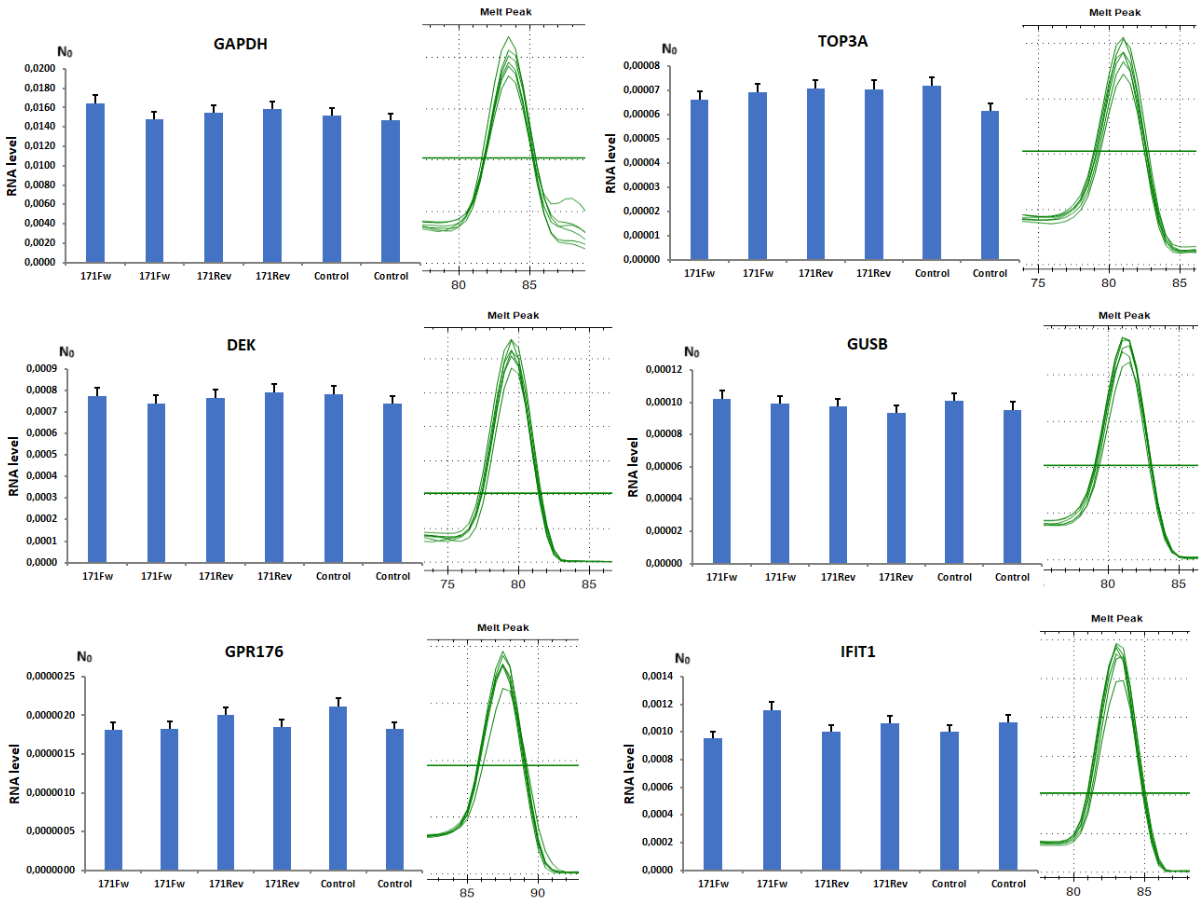

Figure S6. Expression profiles of six housekeeping genes in MJ90hTERT cell line after 24 hr transfection with alpha satellite-expressing vectors (171Fw, 171Rev) and unmodified control vector with corresponding melting curves. Two independent RT-qPCR experiments were performed and averaged values are displayed. Error bars represent standard deviations and averaged N_0 values are expressed in arbitrary fluorescence units. No significant differences in gene expression were observed between transfected samples in all cases (Student's t-test, $P>0.1$).

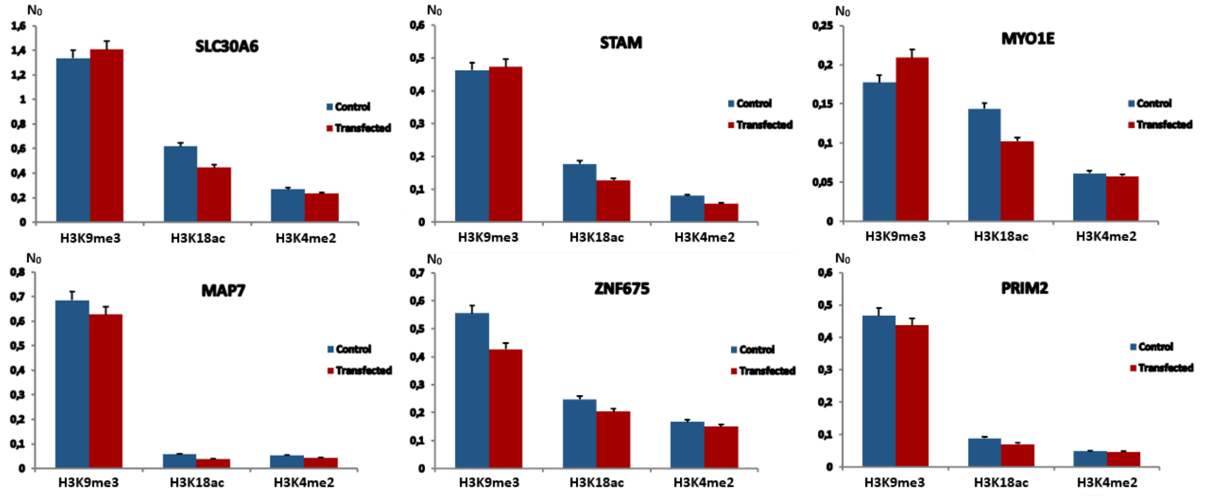

Figure S7. Levels of H3K9me3, H3K18ac and H3K4me2 histone modifications at alpha satellite repeats associated with six genes after 24 hour transfection with 171Rev and unmodified control vectors. Levels of histone modifications were measured by ChIP coupled with quantitative real-time PCR on MJ90hTERT chromatin immediately after each treatment. N_0 values were normalized using N_0 values of input fractions and represent the levels of histone modifications. Columns show averages of two independent experiments and error bars indicate standard deviations. No significant differences with regard to tested histone modifications at alpha repeats in six genes of interest compared to control samples were observed ($P>0.1$ in all cases, Student's t-test).

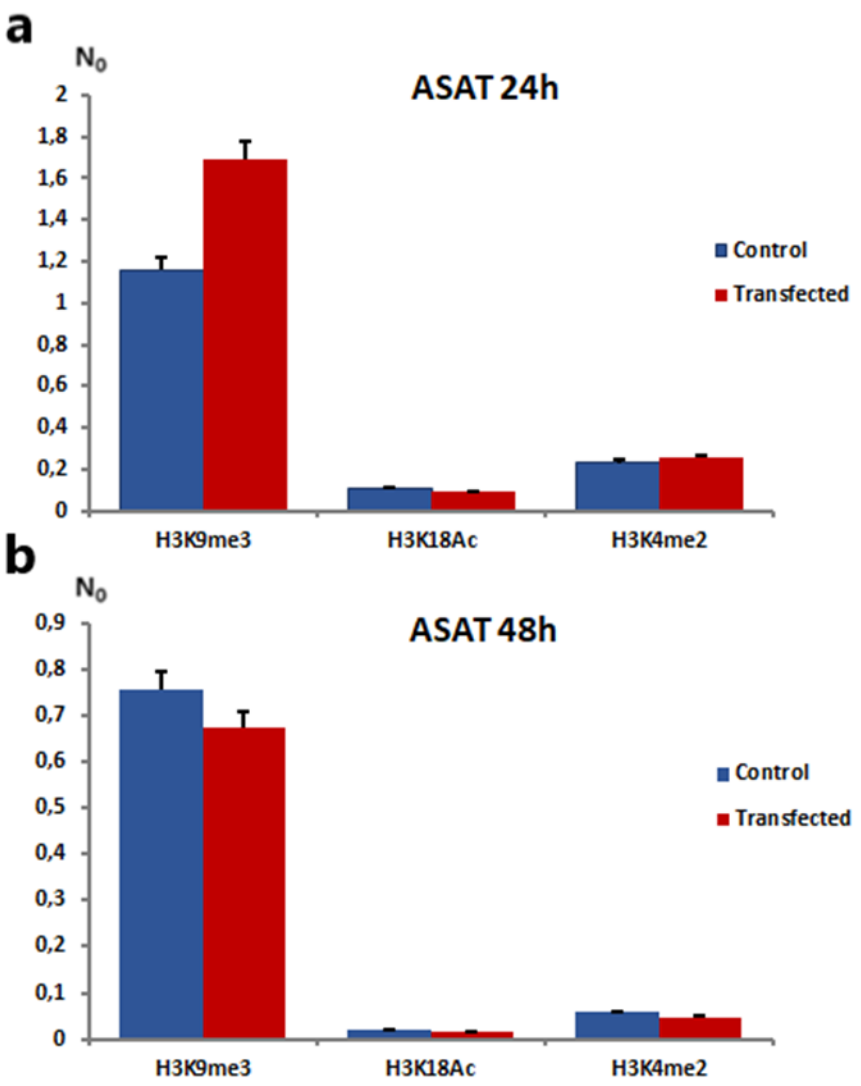

Figure S8. Levels of H3K9me3, H3K18ac and H3K4me2 histone modifications at tandemly arranged alpha satellite repeats after **(a)** 24 hour and **(b)** 48 hour transfection with 171Fw and unmodified control vectors. Levels of histone modifications were measured by ChIP coupled with quantitative real-time PCR on MJ90hTERT chromatin immediately after each treatment. N_0 values were normalized using N_0 values of input fractions and represent the levels of histone modifications. Columns show averages of two independent experiments and error bars indicate standard deviations. H3K9me3 histone modification was upregulated by ≈ 1.5x fold after 24 hour transfection with 171Fw vector compared to control samples ($P<0.05$, Student's t-test). After 48 hour transfection no statistically significant change in H3K9me3 is detected.

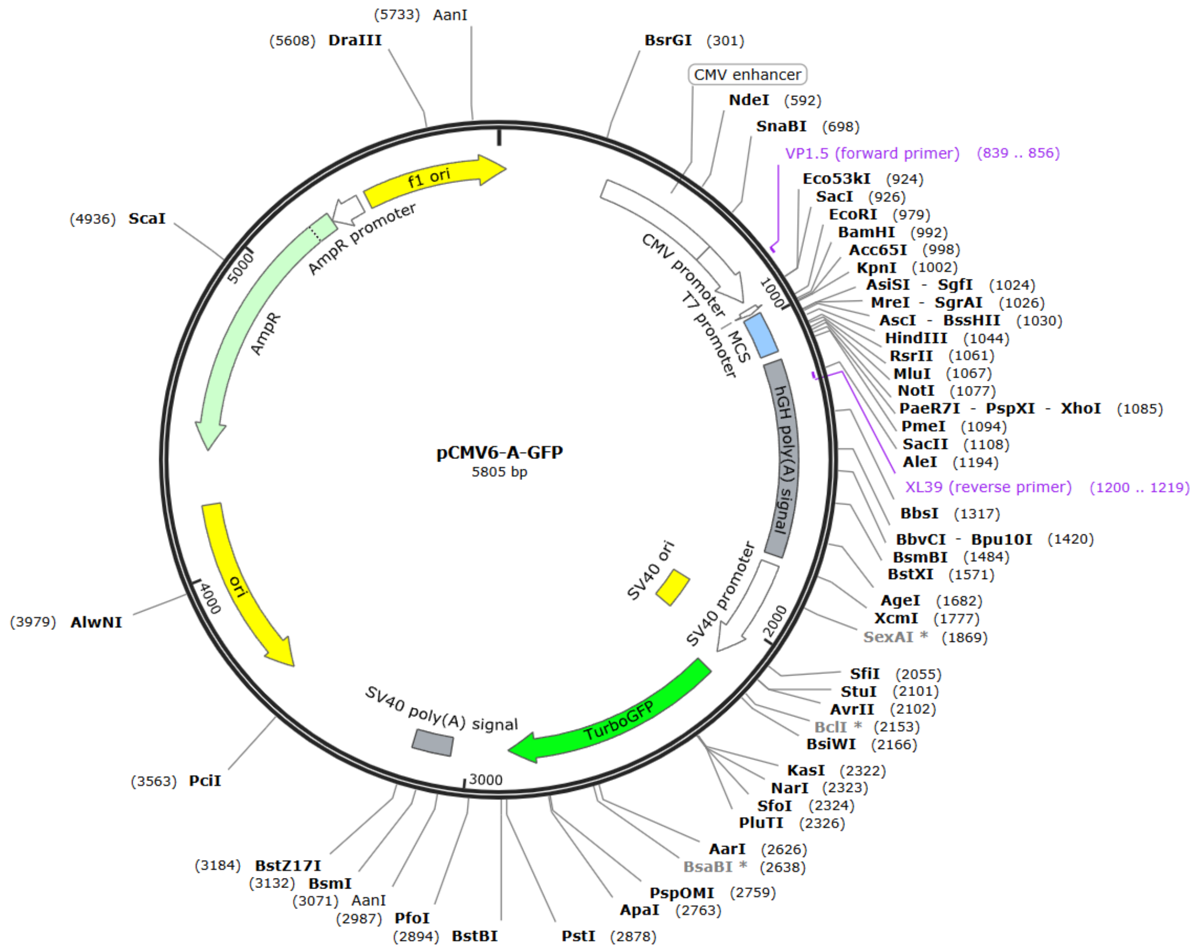

Figure S9. Map of the pCMV6-A-GFP plasmid vector (OriGene) used for transfection. Restriction sites *BamH*I and *Xho*I were selected for ASAT insertion, maintaining the approximately same size of modified and control vectors.
